# Supplementary material for: Childhood vaccination trends among the Maasai nomadic pastoralists: Insights from a community-based vaccine registry in Kenya
Source: PLOS Glob Public Health. 2025 Mar 25;5(3):e0004077. doi: 10.1371/journal.pgph.0004077 (PMC11936188; doi:10.1371/journal.pgph.0004077)
Supplement: S1 Table — (DOCX) [file pgph.0004077.s002.docx]

**S3 Table: Vaccination counts by facility and gender**

| Facility | Number of observations | Observations with a date of birth | Data collection start date | Gender % Female | Gender % Male |
| --- | --- | --- | --- | --- | --- |
| Mara Rianta | 1037 | 1026 | December 2016 | 517 (50.54%) | 506 (49.46%) |
| Talek | 1472 | 1464 | August 2018 | 717 (49.14%) | 742 (50.86%) |
| Ewaso-Ngiro | 3916 | 3843 | April 2016 | 1985 (51.81%) | 1797 (46.91%) |
| Aitong | 2185 | 2154 | July 2016 | 1046 (49.81%) | 1054 (50.19%) |
| Total | 8610 | 8487 |  | 4077 (48.04%) | 4287 (50.51%) |
