# Supplementary material for: Childhood vaccination trends among the Maasai nomadic pastoralists: Insights from a community-based vaccine registry in Kenya
Source: PLOS Glob Public Health. 2025 Mar 25;5(3):e0004077. doi: 10.1371/journal.pgph.0004077 (PMC11936188; doi:10.1371/journal.pgph.0004077)
Supplement: S2 Table — (DOCX) [file pgph.0004077.s003.docx]

**S2 Table: Distribution of vaccinations by facility**

| **Vaccine** | **Dosage** | **Distribution of Immunisations by Facility** | | | |
| --- | --- | --- | --- | --- | --- |
|  | | Mara Rianta | Talek | Ewaso-Ngiro | Aitong |
| **% of records** | | 12.09% | 17.19% | 45.49% | 25.23% |
| BCG | 1 dose | 999 (12.41%)  95 % CI [11.69, 13.15 ] | 1413 (17.55%)  95% CI [16.72, 18.40] | 3555 (44.15%)  95% CI [43.06, 45.24] | 2085 (25.89%)  95% CI [24.94, 26.87] |
| Polio Birth Dose | 1 dose | 721 (16.50%)  95% CI [15.41, 17.64] | 1202 (27.51%)  95% CI [26.19, 28.86] | 1133 (25.93%)  95% CI [24.63, 27.26] | 1313 (30.05%)  95% CI [28.70, 31.44] |
| OPV 1 | 1st dose | 921 (13.47%)  95% CI [12.67, 14.30] | 1234 (18.04%)  95% CI [17.14, 18.98] | 2669 (39.03%)  95% CI [37.87, 40.19] | 2015 (29.46%)  95% CI [28.38 - 30.56] |
| OPV 2 | 2nd dose | 818 (13.50%)  95% CI [12.65, 14.39] | 1131 (18.67%)  95% CI [17.69, 19.67] | 2192 (36.18%)  95% CI [34.97, 37.40] | 1918 (31.66%)  95% CI [30.49, 32.84] |
| OPV 3 | 3rd dose | 713 (13.56%)  95% CI [12.65, 14.52] | 1016 (19.33%)  95% CI [18.27,  20.42] | 1703 (32.39%)  95% CI [31.13, 33.68] | 1825 (34.72%)  95% CI [33.43, 36.02] |
| IPV | 1 dose | 689 (13.33%)  95% CI [12.41, 14.29] | 1025 (19.83%)  95% CI [18.75, 20.95] | 1650 (31.93%)  95% CI [30.66, 33.22] | 1804 (34.91%)  95% CI [33.61, 36.22] |
| DPT 1 | 1st dose | 930 (12.80%)  95% CI [12.04, 13.59] | 1310 (18.02%)  95% CI [17.15, 18.93] | 3004 (41.33%)  95% CI [40.20, 42.47] | 2024 (27.85%)  95% CI [26.82, 28.89] |
| DPT 2 | 2nd dose | 830 (12.88%)  95% CI [12.07, 13.72] | 1230 (19.08%)  95% CI [18.13, 20.07] | 2454 (38.08%)  95% CI [36.89, 39.27] | 1937 (30.05%)  95% CI [28.94, 31.19] |
| DPT 3 | 3rd dose | 732 (13.04%)  95% CI [12.17, 13.95] | 1114 (19.85%)  95% CI [18.81, 20.91] | 1935 (34.48%)  95% CI [33.24, 35.74] | 1831 (32.63%)  95% CI [31.40, 33.87] |
| PCV 1 | 1st dose | 927 (12.76%)  95% CI [12.00, 13.55] | 1309 (18.02%)  95% CI [17.14, 18.93] | 3002 (41.33%)  95% CI [40.20, 42.48] | 2025 (27.88%)  95% CI [26.85, 28.93] |
| PCV 2 | 2nd dose | 824 (12.78%)  95% CI [11.98, 13.62] | 1230 (19.08%)  95% CI [18.13, 20.07] | 2454 (38.08%)  95% CI [36.89, 39.27] | 1937 (30.05%)  95% CI [28.94, 31.19] |
| PCV 3 | 3rd dose | 728 (12.98%)  95% CI [12.10, 13.88] | 1110 (19.78%)  95% CI [18.74, 20.85] | 1943 (34.62%)  95% CI [33.38, 35.88] | 1831 (32.63%)  95% CI [31.40, 33.87] |
| Rota 1 | 1st dose | 857 (13.60%)  95% CI [12.77, 14.47] | 1180 (18.73%)  95% CI [17.77, 19.72] | 2395 (38.02%)  95% CI [36.82, 39.23] | 1868 (29.65%)  95% CI [28.52, 30.80] |
| Rota 2 | 2nd dose | 721 (13.42%)  95% CI [12.52, 14.36] | 988 (18.38%)  95% CI [17.36, 19.45] | 1895 (35.26%)  95% CI [33.98, 36.56] | 1770 (32.94%)  95% CI [31.68, 34.21] |
| Vit. A | 1st dose | 432 (11.44%)  95% CI [10.45, 12.50] | 936 (24.80%)  95% CI [23.42, 26.20] | 845 (22.38%)  95% CI [21.06, 23.75] | 1562 (41.38%)  95% CI [39.80, 42.97] |
| MR 1 | 1st dose | 446 (12.15%)  95% CI [11.11, 13.25] | 735 (20.03%)  95% CI [18.74, 21.36] | 1061 (28.91%)  95% CI [27.45, 30.41] | 1428 (38.91%)  95% CI [37.33, 13.88] |
| MR 2 | 2nd dose | 167 (12.88%)  95% CI [11.10, 14.82] | 275 (21.20%)  95% CI [19.01, 23.53] | 178 (13.72%)  95% CI [11.90, 15.72] | 677 (52.20%)  95% CI [49.44, 54.95] |
