# Supplementary material for: Childhood vaccination trends among the Maasai nomadic pastoralists: Insights from a community-based vaccine registry in Kenya
Source: PLOS Glob Public Health. 2025 Mar 25;5(3):e0004077. doi: 10.1371/journal.pgph.0004077 (PMC11936188; doi:10.1371/journal.pgph.0004077)
Supplement: S3 Table — (DOCX) [file pgph.0004077.s004.docx]

**S4 Table: Dropout rate by dosage and facility**

|  | OPV | | DPT | | PCV | | Rota | MR |
| --- | --- | --- | --- | --- | --- | --- | --- | --- |
|  | 1st-2nd dose | 2nd-3rd dose | 1st-2nd dose | 2nd-3rd dose | 1st-2nd dose | 2nd-3rd dose | 1st-2nd dose | 1st-2nd dose |
| Mara Rianta | 11.18% | 12.84% | 10.75% | 11.81% | 11.11% | 11.65% | 15.87% | 62.56% |
| Talek | 8.35% | 10.17% | 6.11% | 9.43% | 6.04% | 9.76% | 16.27% | 62.59% |
| Ewaso-Ngiro | 17.87% | 22.31% | 18.31% | 21.15% | 18.25% | 20.82% | 20.88% | 83.22% |
| Aitong | 4.81% | 4.85% | 4.30% | 5.47% | 4.35% | 5.47% | 5.25% | 52.59% |
| Overall | 11.41% | 13.24% | 11.24% | 13.01% | 11.26% | 12.92% | 14.70% | 64.66% |
