# Supplementary material for: Childhood vaccination trends among the Maasai nomadic pastoralists: Insights from a community-based vaccine registry in Kenya
Source: PLOS Glob Public Health. 2025 Mar 25;5(3):e0004077. doi: 10.1371/journal.pgph.0004077 (PMC11936188; doi:10.1371/journal.pgph.0004077)
Supplement: Attachment — Submitted filename: Response to reviewers.docx [file pgph.0004077.s007.docx]

**Response to Reviewers**

| **REVIEWER 1** | |
| --- | --- |
| **Reviewer’s Comment** | **Author’s Response** |
| A well written paper that contributes to framing a more comprehensive profile of Kenya's routine immunization coverage, timeliness and dropout rates. | We appreciate the reviewer's feedback and the opportunity to enhance the clarity and interpretative aspects of our manuscript. |
| A few of the sentences could benefit from being rewritten for clarity. An example can be found in line 325 in the sentence starting with 'With limited access to routine vaccines, children become more vulnerable...' | Agreed.  We have carefully reviewed the manuscript and have revised the sentence for clarity.   Please see lines 330 - 336  We have also reviewed the rest of the manuscript to ensure that other sentences are equally clear and concise. |
| Another example is from line 172 to 174. While a trained person would know that '...the difference between consecutive doses...' is referring to *the difference between children receiving consecutive doses*, this can be stated to aid clarity. | Thank you for highlighting this point. We have clarified the methodology for calculating dropout rates in the manuscript to ensure it is more explicit. The revised description now reads:  *"For multi-dose vaccines, dropout rates were calculated by subtracting the number of children who received the subsequent dose (e.g., dose 2 or dose 3) from those who received the preceding dose (e.g., dose 1 or dose 2), and dividing this difference by the number of children who initially received the preceding dose.*  Please see lines 179 - 182 |
| **REVIEWER 2** | |
| Thank you for this interesting article about an important topic in a minority population. I hope that the following suggestions would help to refine this paper. | We appreciate your thoughtful feedback and recognition of the importance of our topic. |
| - Title: Subtle change, but I would call it “community-based” - given that as far as I have understood from your manuscript, the process of keeping local vaccination records is national policy, using a national template. It is just that individual sites do not share their data nationally. If I have misunderstood this, then it should be better clarified in the article. | Agreed.  The updated title now reads: “Childhood vaccination trends among the Maasai nomadic pastoralists: Insights from a community-based vaccine registry in Kenya”  Please see lines 1 -2  More clarity on the framework guiding record-keeping and the gap in data sharing have been highlighted in the manuscript. |
| Data sharing: There is a note to say that the data will be made available with an email address to contact - but I do not believe this is allowed under the journal policies. | We recognize the PLOS data-sharing policy requires that all data be made fully available without restriction. We have updated the data availability section to reflect this and provide the relevant contact information and links for those who wish to obtain the data. |
| Appropriateness of statistical analyses: Not enough detail has been given in some of the analyses for me to have a clear idea of whether the methods were appropriate. | We have revised the manuscript to provide more comprehensive details on the statistical methods used   Please see our Methods section |
| Abstract: - The number of villages in the abstract is different from the number in the main results section of the paper. - The headline results presented in the abstract are not presented in the main results section of the paper. | We have updated our abstract to align with our results.  Please see lines 33 - 46 |
| The background section currently is around 85% about the global childhood vaccination situation, and only 15% about what is happening in Kenya (with nothing in the background about Narok). To be an effective paper, it really needs to be the other way around, with a real emphasis on the communities you are studying. You really need to give a lot more context specific to the local area, as well as the rationale for undertaking this particular study (in this particular area and with these particular clinics) and its anticipated contribution. The word “Maasai” was not even mentioned once in the background section! - Please give us much more detail on the population within this study area! For example, are they are homogenous ethnic group (all Maasai) and what is the age distribution? Is there any indication of the size of the annual birth cohort?  The words “nomadic pastoralists” was mentioned in the title, but not anywhere else in the article! Readers are from around the world, where there many different kinds of nomadic pastoral lifestyles - therefore there needs to be better description of what this term means in the context of the Maasai. What specific challenges does this pose for childhood immunisation? | We have revised the background section to focus more on the local context, specifically the Maasai population in Narok and the vaccination context in Kenya.  Please see lines 103 - 143 |
| You were discussing access to clinics only in terms of distance - how about other factors like quality of roads, transport options, any specific physical or manmade barriers to travel? | Agreed.  While our study primarily focused on distance as a measure of accessibility, we recognize that other factors such as road quality, availability of transport options, and potential physical or manmade barriers (e.g., rivers, mountains,) are crucial to a comprehensive understanding of access to care.  At present, we’ve acknowledged these limitations and emphasized the need to consider these additional variables in future research efforts.  Please see lines 350 – 357. |
| Are there any costs associated with childhood vaccines? Even if the vaccines themselves are free, do parents need to pay a small service fee to be seen at the clinic?  Are these clinics the only way for children to receive childhood vaccines? Or are there community healthcare workers who visit door to door? | In Kenya, childhood vaccines are provided free of charge through the national immunization program at public health facilities. While the vaccines themselves are free, families may still face indirect costs, such as transport expenses to reach the clinic, especially in rural areas. In public facilities, there is typically no service fee associated with vaccinations. However, some private clinics may charge a small fee for the service, though this is not the case in government-run facilities.  In addition to clinics, community health workers play a vital role in delivering vaccines, especially in hard-to-reach areas. These workers often visit homes and conduct door-to-door immunization campaigns, which help increase vaccine coverage among populations that may face barriers to accessing healthcare facilities. Such outreach programs are particularly effective in areas with poor infrastructure, where geographic and financial barriers make clinic visits more challenging.  We have addressed these nuances in the discussion and highlighted the importance of both clinical services and community-based outreach in ensuring equitable access to vaccinations. |
| Was this analysis part of a bigger project on improving immunisation coverage in this region? The context of data collection for this study could also be clarified. | Our analysis was conducted as part of a broader effort to understand community-level trends in immunization coverage using data from community-based health information systems. While not directly linked to a specific project aimed at improving immunization coverage, this study provides critical insights into local vaccination patterns and access barriers, which could inform future interventions.  Please see lines 130 - 143 |
| It would be good to know more about the training received by the clinic staff - you had mentioned that there were errors in record keeping. | While our study did not directly assess the level of training provided to staff, we recognize that gaps in training or ongoing support may contribute to errors in data collection and reporting. |
| Where you’ve said “most prevalent in the first year of life” - firstly, “prevalent” is probably not the right word to use here, but difficult to know without knowing what evidence this sentence is based on. Do you have evidence to support this sentence? | We have revised the sentence for clarity and added the supporting citations.  Please see lines 156 - 161 |
| (I fully recognise that it would take a lot of words to answer some of the following questions - for the sake of the word count, maybe it would be useful to include some of these explanations in the supplementary information.) - Good use of subheadings to help readers keep track of the different considerations that went into the design of this study. - While you have given the dates when you were extracting records, you have not given the exact period that the records date from. Do we assume that these are the complete immunisation records of each clinic dating back to when they first started record keeping until a certain date in 2024? | We appreciate your suggestion regarding the use of supplementary information for more extensive explanations.  Please see our supplementary file. |
| - Have you left a gap in terms of the period of analysis and the period of data availability? In other words, if you collected data until June 2024, then do you analyse data on vaccine doses due until maybe March 2024, so that you have 3 months to see if the child will be late or might drop out altogether? | We have included additional analyses in the supplementary files to provide further insights. |
| - You mentioned there were errors or other quality issues in the record keeping. Was there a checklist / a list of criteria that you used to judge the quality of a record - and did you exclude any records that did not meet minimum data quality standards? | While we did not have a formal checklist, we assessed the quality of the records based on criteria such as completeness, accuracy, and consistency of the data entries.  Any records that fell short of these minimum data quality standards were excluded from our analysis. |
| - Digitalisation of the data collected was mentioned - how exactly was this done? Were paper records just scanned into digital images? Was the data fed into a study database? Does this digitalised data get shared back to these community clinics? | We transferred paper records into Excel sheets, which allowed us to systematically organize and analyze the information. This process involved manually entering the data to ensure accuracy.  This process was jointly conducted with our in-country partners. |
| The serial number assigned to each child, is this number used across the 4 clinics? In other words, if a child moves from one area to another and receives their vaccines from two different clinics within the 4 that are included in the study, could you track this child from one clinic to the next based on their serial number? - Where are the date of birth and other personal details obtained from - directly from the mother and child booklet? - How reliable are the dates of birth - given that they are the basis of many of the calculations in this paper? Were there records where the date of birth was missing, and what was done with these records? Were they excluded from analysis? | The serial number assigned to each child is unique to the individual clinic and does not extend across multiple clinics. If a child receives vaccines at different clinics, tracking them would require cross-referencing personal identifiers like name and date of birth.  Dates of birth were obtained from the mother-child health book, a reliable source verified by health workers during registration. However, any records with missing or ambiguous dates of birth were excluded from the analysis to maintain accuracy.  This has been clarified in the manuscript.  Please see lines 224 - 228 |
| Did the analysis of timeliness take into account the interval after the previous dose? So for example, according to your table, somebody could get PCV2 at 97 days of age and be counted as “on time”, but then they can get PCV3 at 99 days of age and be counted as “on time” again. But an interval of 2 days between these 2 doses would definitely not produce the optimal immune response! This would be a lot of work to review the timing of vaccine doses for each individual child, so it is perfectly reasonable if this degree of analysis was not done - but it needs to be mentioned as a limitation to the study.  Similarly, do you account for any catch-up timetables for children with delayed vaccination? | Timeliness by vaccine dose was calculated by subtracting the date of birth from the date of vaccination, with the age in days compared to the WHO and Kenyan Ministry of Health recommended immunization schedules.  We recognize that short intervals between doses, as in your example, could impact the optimal immune response, but this level of detailed review was beyond the scope of our analysis.  Specific catch-up schedules for those who were delayed were not differentiated in the dataset. |
| - How did you deal with any discrepancies between what was in the clinic immunisation register and the mother-and-child booklet? Which of these was used as the “source of truth” where there are discrepancies? | In cases where there were discrepancies between the clinic immunization register and the mother-and-child booklet, the clinic immunization register was considered the "source of truth." This decision was based on the assumption that clinic records are maintained by healthcare professionals and are likely to reflect more accurate and systematically recorded data. |
| - What was the method used to obtain 95% confidence intervals? | The 95% confidence intervals were calculated using the Wilson score method. This method is particularly effective for estimating confidence intervals for proportions, especially when sample sizes are small.The Wilson score method adjusts for potential bias in the estimation of proportions, providing more reliable intervals |
| - What are the date criteria for MR dose 1 and 2 in terms of the number of allowable days before it counts as “delayed”? Those cells in the table are blank. | The recommended age for **MR dose 1** is 9 months, and for **MR dose 2**, it is 18 months, in line with national immunization schedules.  We will update the table to reflect these criteria and ensure that no cells remain blank. |
| - Four primary healthcare clinics: are they they only primary healthcare clinics in this region? What is the population size that they serve? What proportion of this population are children? What are the difference between these clinics - if any? What are the differences in the characteristics of the people served by each clinic? Is the patient base of one clinic more nomadic/transient than others? | The primary healthcare clinics in our study are the largest community-based service providers in the region, though they are not the only clinics available.  While we do not have exact figures for the population size they serve, they collectively support a significant portion of the regional population, including a large number of children under five years old. |
| - With the 8487 records, is each record 1 child (with all their vaccines included)? Or is each record 1 line in the immunisation register (in which case each child may have more than 1 record)? This really needs to be made clear. | To clarify, each record corresponds to a unique child, representing their vaccination history, which may include multiple entries for different vaccines received.  Please see lines 199 - 207 |
| - It would be useful to have a descriptive statistical analysis of data obtained from each of the 4 clinics: how many records from each clinic? How many vaccine doses were given at each clinic? | We will include information such as the number of records from each clinic and the total vaccine doses administered at each clinic.  Please see our supplementary table 2. |
| Figure 2 shows a sharp but transient improvement in 3rd dose coverage for all 3 vaccines in year 2020. Why was this? The paper should explain. | Kindly note that Figure 2 only shows the trends for the first and third dose of DPT. This is the vaccine used for assessing coverage.  We have provided an explanation of the observed trends in the manuscript. |
| -- The 1st paragraph of results said that there were 258 villages represented in the data. However, there are clearly fewer than 258 individual dots in each of the 6 maps. It would be useful to include a footnote explaining which villages were excluded from the maps - was it because there was insufficient data from the village? And if so, what was the cutoff used to decide whether a village had enough data to display? | We have enhanced our figures to include villages with incomplete data. |
| -- The locations of the community clinics are not displayed on the map - therefore readers cannot see the relationship between distance from the clinic with any outcomes. | We will revise the maps to include the locations of the community clinics.  Please see our revised Figure 1. |
| - Vaccine coverage by village: if I have understood correctly, then there are 8487 children from 258 villages. This means that there are on average 33 children per village. And then this number is sub-divided by year when you calculate percent coverage each year, which means that on average there are probably fewer than 10 children per village who are due for this particular antigen and could be counted in the denominator. It is not appropriate to calculate percentage coverage with such a small denominator, or to draw conclusions at the village level. I would recommend working with a larger geographical unit - if possible, so that there are at least 100 individuals in the denominator. | Thank you for your insightful comment.  Vaccine antigen coverage was computed using a service-based denominator, which reflects the total number of children who presented at the facility. Specifically, the denominator comprised all children who had received the BCG vaccine and were expected to return for follow-up immunizations.  Please see lines 229 - 232 |
| - Timeliness: you had said the *highest* decline between the first and 3rd doses was observed in DPT, PCV and OPV - but given that these are the only 3-dose vaccines that are included in this study, the word “highest” should be removed.  - Figure 4: Are there equal numbers of children in each category? It is difficult to be able to draw conclusions/exclude statistical artefacts from this figure without knowing whether there may be hundreds of children in the category closest to the clinic but only dozens of children in the furthest category. | Agreed.  We have revised our draft to reflect that the decline between the first and third doses was observed in these vaccines without implying a comparison.  The vaccination rates are different for each vaccine antigen. Understanding these trends is primarily why we conducted the study. |
| - Were the children in the “delayed” group mutually exclusive from children in the “dropout” group? So you waited for a certain length of time after the vaccine was due, and if they had not received the vaccine by then, they counted as “dropout”? The methodology of classifying children as “delay” or “dropout” needs to be clear. | The methodology we used to classify children into these categories was based on the guidelines/thresholds provided by the World Health Organization (WHO) and the Kenyan Ministry of Health regarding vaccination timeliness.  We have clearly described this methodology in the manuscript. |
| - You have not made any comments on zero-dose (beyond initial BCG) children. What percentage of children received a BCG vaccine as part of their “enrolment” in the clinic denominator, but then never received any subsequent vaccines? | Zero-dose children, as defined by the Immunization Agenda 2030 (IA2030), are those who lack access to or are never reached by routine immunization services. Operationally, these are children who did not receive their first dose of a vaccine containing diphtheria-tetanus-pertussis (DTP). |
| You have recommended to use “the most granular geographic delineations possible.” Would knowing somebody’s address to the nearest 100m result in a significant difference to public health measures compared to knowing their address to the nearest 1km? | While more granular data may enhance localized decision-making, such precision is not always necessary for broader public health strategies, where resource allocation and infrastructure development are based on more regional trends.  Ideally, the focuses should be on geographical delineations that align with the scale at which health policies are typically implemented for this community. |
| - Drought is described as a potential contributor to vaccination delays, with evidence that this is the case in sub-Saharan Africa. However, you have not linked this to the specific communities you are describing. Did they suffer droughts during the period of time that your analysis covered? And if so, did it affect the entire community equally, or was it more obvious in the records of one clinic? | In our analysis, the Maasai community, known for their nomadic lifestyle, is particularly affected by drought as they move in search of water and pasture. Seasonal rainfall patterns dictate these movements, which can lead to uneven access to vaccinations across different clinics. While we did not specifically track drought events during our study period, the mobility of the Maasai likely influenced vaccination records.  Please see 359 - 367 |
| - The results (especially the supplementary material) have shown that many doses of vaccines are given early - especially the vaccines at the 6-week-old time point, where more vaccines are given early than late. However, this is not mentioned at all in the discussions. This is quite a glaring omission, and it would be useful to know why so many doses of vaccines might be given early. | Early vaccinations may occur due to various factors, including caregiver misconceptions about optimal timing, health worker practices, or the availability of vaccines at the clinics.  Please see our enhanced explanation in lines 369 – 373. |
| - Are there any particular health impacts observed in Narok that might be due to a lower childhood vaccination coverage compared to the rest of the country? | Lower vaccination rates lead to a higher prevalence of diseases such as measles, polio, and whooping cough, resulting in increased hospitalizations and mortality among children. Children who contract vaccine-preventable diseases may suffer from long-term health issues, further complicating their healthcare needs and increasing the burden on local health systems. Additionally, missed immunization opportunities may hinder their education and future productivity, perpetuating cycles of disadvantage.  Our study did not explore/compare the direct impacts of low vaccination coverage in Narok and the rest of Kenya as we consider every context to be epidemiologically unique. |
| - You had mentioned supply issues - were there any specific interruptions to supply during the time covered by the study? And if yes, was there an observable impact on local vaccine coverage at these 4 clinics? | During the COVID-19 pandemic, vaccine supply interruptions significantly affected local coverage at the four clinics in Narok County. Delayed shipments and reduced availability of certain vaccines led to gaps in immunization schedules, while health resources were often redirected to address the pandemic. As a result, many children missed their routine vaccinations.  Please see our explanation in lines 310 - 319 |
| - You mentioned transition out of Gavi support - it might be useful to give the reader an idea about where Kenya is exactly in this trajectory, and the feasibility of adequate domestic manufacturing. | Agreed.  Please see lines 306 - 309 |
| - You may want to shift the recommendation for electronic immunisation records until later in the discussion section. At the moment, it comes across as a very sudden idea that is not linked to any other ideas - and it is only until later, when you discuss data entry errors or poor legibility that it becomes obvious why you are suggesting this. It might be useful to move the recommendation to use electronic records after the explanation about poor data quality. | Thank you for your suggestion. We agree that it is important to clarify upfront that the results are based on data from community-based registries. This will provide clearer context for readers regarding the data quality issues and will support the subsequent recommendation for transitioning to electronic immunization records.  We will revise the manuscript to ensure this point is clearly stated earlier in the discussion. |
| Conclusion: - Succinct conclusion, no specific comments other than that it might be useful to think about how the results of this study can be used to train vaccination providers at these clinics. | We have revised the conclusion to highlight how the results of this study can be applied to train vaccination providers at these clinics, where two of our authors are currently working on the practical implementation of the study’s findings. |
| Overall: - Presenting the data only as proportions and not as absolute counts means that the reader cannot fully interpret the data that they are seeing. It would be very helpful for your paper to have data presented both ways. | We will revise the manuscript to include both absolute counts and proportions in the supplementary section to enhance data interpretability for the reader.  Please see supplementary table 3 and table 4 |
