# Supplementary material for: Childhood vaccination trends among the Maasai nomadic pastoralists: Insights from a community-based vaccine registry in Kenya
Source: PLOS Glob Public Health. 2025 Mar 25;5(3):e0004077. doi: 10.1371/journal.pgph.0004077 (PMC11936188; doi:10.1371/journal.pgph.0004077)
Supplement: Attachment — Submitted filename: Response to reviewers2.docx [file pgph.0004077.s009.docx]

**Response to Reviewers**

| **REVIEWER 2** | |
| --- | --- |
| **Reviewer’s Comment** | **Author’s Response** |
| Thank you for all your revisions, and for your very helpful answers to my previous questions!  However, not all the points that you had said you would address in the manuscript made their way into the final manuscript. For example, there was mention of supplementary table 3 and 4 that were meant to have been added, but did not make their way to the final manuscript. It might be useful to review the final manuscript in parallel with your responses to the reviewer to make sure that all planned edits had been incorporated. | Thank you for your thorough review and feedback.  The revised manuscript and supplementary materials will be updated accordingly.  Please see Supplementary table 1 – 3. |
| The other major area to focus on is the denominator for the coverage estimates - making sure that this is as clearly explained and as transparently reported as possible. | The standard approach to computing annual vaccine coverage for various antigens is by calculating the proportion of children who receive a vaccine relative to the total projected population of children under 1 year (denominator). However, the effectiveness of this method for a community-based vaccine registry is hindered by inaccurate estimates of the target denominator population size and a lack of accounting for migrations and health facility catchments.  An alternative method that seeks to overcome the challenges of population-based coverage exploits linked health service data, e.g., other vaccines, as the denominator as shown from previous studies[1,2].  Our study thus focussed on “within reported data” coverage and carefully selected all children who received the BCG vaccine and were expected to return for follow-up immunizations as the denominator.  Additional references have been added. Please see lines 186 – 189. |
| Background:  Great work on your edits. This now helps me (and readers) understand the context so much better, and also enriches the paper. Thank you! | Thank you for the kind feedback! |
| Methods:  • Thank you for clarifying that the clinic record was regarded as the “source of truth” in cases of discrepancies between the two sources - it would also be good to put this into the paper  • Table 1: Thank you for filling in the table with some date criteria for MR, but I wonder if there are errors.The “delayed” criterion for MR1 is not possible, given that it occurs earlier than the “early” criterion. No “delayed” criterion for MR2.  Important: the entire table needs referencing | Agreed.  We will include the clarification regarding the clinic record as the "source of truth" in the Methods section.  Please see lines 157 – 160.  For Table 1, we will correct the MR1 delayed criteria to resolve the inconsistency.  Regarding MR2, it can be administered at any time beyond the 18 months and does not have a "delayed" criterion.  Please see the edited Table 1 |
| Results:  • Thank you for including the actual dataset in the supplementary material - now I think I can see your methods for some of the coverage calculations that I had previously queried. I think that to make your results absolutely clear, the following points need to be included:   - State that the 8487 records is your overall denominator: Each of these records corresponds to a child who has had a dose of BCG or who has migrated into the catchment of one of these clinics, and each child may have 1 or more vaccines recorded (this was what I had wanted when I had asked for absolute clarity in my previous review about whether each record refers to 1 child or 1 vaccine dose - it makes a huge difference to your sample size!) - State whether there was any adjustments to the denominator to account for children who migrated into the area, or whether there was just an assumption that migration in roughly equated to migration out | Agreed.  We have clarified that the 8,487 records represent the overall denominator, with each record corresponding to a child who has received a BCG dose or migrated into the clinic catchment, and that each child may have one or more vaccines recorded.  No adjustments for incomplete reporting was done to estimate the expected number of total pregnancies, deliveries, births, and infants as in the case of the census-based approaches.  Please see lines 162 – 166, lines 206 – 211. |
| - Thank you for letting me know in your response that you did exclude some records due to data quality. Given that this is your denominator and is crucial to your calculation of coverage, it would be good to also include this information in the manuscript itself, and an indication of how many records were excluded from the denominator due to poor data quality. - Given that you are reporting annual coverage, you need a separate table outlining the denominator you used for each year | We have included information in the manuscript about records excluded due to data quality, detailing the number excluded by facility and vaccine.  Please see supplementary table 2. |
| o Given that you had to adjust the denominator for each dose of each vaccine to account for eligibility, it would be useful to add another table to the supplementary material listing the actual denominator used to calculate coverage for each dose of each vaccine - this ensures reproducibility of your results | Agreed.  Please see supplementary table 2 |
| • It would be useful to clarify whether all children at each clinic were of Maasai background, or only a proportion | While the region is predominantly Maasai (2019 Census), we do not have specific data on the ethnic background of each child attending the clinics, as each child was referenced only by a unique ID and village. Therefore, we cannot confirm whether all children are of Maasai background or only a proportion. |
| • I had previously made the comment: “Vaccine coverage by village: if I have understood correctly, then there are 8487 children from 258 villages. This means that there are on average 33 children per village. And then this number is sub-divided by year when you calculate percent coverage each year, which means that on average there are probably fewer than 10 children per village who are due for this particular antigen and could be counted in the denominator. It is not appropriate to calculate percentage coverage with such a small denominator, or to draw conclusions at the village level. I would recommend working with a larger geographical unit - if possible, so that there are at least 100 individuals in the denominator.”  o I can see that you had responded to this comment - however, I think you may not have understood my original point. I do not see problems with sample size when you look at all 8487 records as a whole. My comment only applies to Figure 3 and related analyses: when you break this number down by village AND year AND specific vaccine dose, then I doubt you have an adequate sample size to really support any firm conclusions being drawn at this village level.   I think to keep this part of the analysis in the paper, it would be important to highlight that due to small sample sizes at the village level, this is the trend that the data appears to show | Please review our corrected manuscript.  The analysis included 8487 vaccination records for children from 176 villages spread across different sub-counties in Narok.  Vaccination coverage was stratified by year and village to show the different trends.  Coverage for each vaccine antigen was calculated as the number of specific vaccines administered in a given year and village, divided by the denominator.  The small sample sizes (low coverage) may indicate suboptimal vaccine coverage and immunity gaps, underscoring the need for targeted catch-up efforts in specific regions and communities. This motivated our analysis of trends using a community-based registry.  Furthermore, an adequate sample size or threshold remains undefined for this community, and our estimates may be biased due to inaccuracies in the numerators and denominators, arising from record-keeping errors, unaccounted migrations, and variations within health facility catchments.  Please see our study limitations. |
| Discussion:  • The added comment about the context of supply / funding is very useful - thank you! | Thank you for your feedback! |
| • It would be useful to comment on the fact that these communities show a similar trend to pretty much everywhere in the world - that coverage drops in subsequent doses!  o But this also means that a useful recommendation is to learn from other systems around the world about the strategies used to improve coverage of subsequent doses | Agreed.  We have noted that, like many places globally, coverage drops in subsequent doses. We have also recommended learning from global strategies to improve coverage for later doses.  Please see 92 -98 |
| • Limitations:  o You need to make it clear that one limitation in your analysis of geographical trends is that you have not explored whether there are other explanations for what you had observed: for example, could it be that people living in more peripheral villages have a more nomadic lifestyle than those living closer to clinic locations? Could it be that people in the peripheral areas just went over the border to receive vaccines in a county not covered by this study? Could it be that peripheral villages have a smaller population, and so the presence of one under-vaccinated child can cause a greater fluctuation in the data (and therefore the findings can be attributed to statistical artefact)?   In other words, access to clinics may not be the ONLY reason why the coverage appears to be lower in the more distant villages - there may be a range of other factors  o Another limitation: using receipt of BCG vaccine as a denominator, which means that the children who do not receive a birth dose BCG at all are excluded from this study  o Thank you for all your responses to my questions during the previous review. Your responses also prompted the need to include another limitation: that these clinics are not the only source of vaccines in the county, and therefore there may be a small possibility that children who were recorded as having “missed” doses at these clinics may have received these doses through other channels. This issue of the clinics not capturing all doses is also very important to consider in light of describing a highly mobile population - are these children necessarily confined to this particular county with its 4 sub-county clinics for their vaccines?  o Another limitation from responses: that the study does not look at the interval between 2 individual doses in its assessment of “early” or “delayed” - as I have said previously, a reasonable decision to make based on human/time resources, but just needs to be mentioned in the limitations | Agreed.  Our analysis was conducted as part of a broader effort to understand community-level trends in immunization coverage using data from community-based health information systems. The findings have important implications for efforts to track maternal health indicators in individual communities using their own data.  Please see lines 343 – 370. |
| Minor edits:  • Check for any unnecessary capitalisation in the manuscript - for example, “Pneumococcal Conjugate Vaccine” | This has been addressed in the revised manuscript. |
| Referencing:  [Editors, I just wanted to check a couple of things:  • Ref #33 - have enough details been provided in the bibliography?  • Shapefile for map: the source of the base map was given in the figure itself. Does this also need to be included in the bibliography?] | Agreed.  This has been corrected in the manuscript. |
